# Oxidative Modification of Proteins in Pediatric Cystic Fibrosis with Bacterial Infections

**DOI:** 10.1155/2014/389629

**Published:** 2014-04-03

**Authors:** Izabela Sadowska-Bartosz, Sabina Galiniak, Grzegorz Bartosz, Marta Rachel

**Affiliations:** ^1^Department of Biochemistry and Cell Biology, University of Rzeszów, Zelwerowicza 4, 35-601 Rzeszów, Poland; ^2^Department of Molecular Biophysics, University of Łódź, Pomorska 141/143, 90-236 Łódź, Poland; ^3^Institute of Physiotherapy, Faculty of Medicine, University of Rzeszów, Warszawska 26a, 35-205 Rzeszów, Poland

## Abstract

*Pseudomonas aeruginosa* and *Staphylococcus aureus* cause chronic lung infection in cystic fibrosis (CF) patients, inducing chronic oxidative stress. Several markers of plasma protein oxidative damage and glycoxidation and activities of erythrocyte antioxidant enzymes have been compared in stable CF patients chronically infected with *Pseudomonas aeruginosa* (*n* = 12) and *Staphylococcus aureus* (*n* = 10) in relation to healthy subjects (*n* = 11). Concentration of nitric oxide was also measured in the exhaled air from the lower respiratory tract of patients with CF. Elevated glycophore (4.22 ± 0.91 and 4.19 ± 1.04 versus control 3.18 ± 0.53 fluorescence units (FU)/mg protein; *P* < 0.05) and carbonyl group levels (1.9 ± 0.64, 1.87 ± 0.45 versus control 0.94 ± 0.19 nmol/mg protein; *P* < 0.05) as well as increased glutathione *S*-transferase activity (2.51 ± 0.88 and 2.57 ± 0.79 U/g Hb versus 0.77 ± 0.16 U/g Hb; *P* < 0.05) were noted in *Pseudomonas aeruginosa* and *Staphylococcus aureus* infected CF. Kynurenine level (4.91 ± 1.22 versus 3.89 ± 0.54 FU/mg protein; *P* < 0.05) was elevated only in *Staphylococcus aureus* infected CF. These results confirm oxidative stress in CF and demonstrate the usefulness of the glycophore level and protein carbonyl groups as markers of oxidative modifications of plasma proteins in this disease.

## 1. Introduction

Cystic fibrosis (CF) is a genetic systemic disease, involving the disorder of secretion of exocrine glands (production of a too sticky mucus), causing changes in the respiratory and digestive tracts. A well-characterized genetic cause of this recessive disease is one of 1,500 mutations in the CF transmembrane conductance regulator (CFTR) including the most common known one as ΔF508. Although the gene mutations cause a number of clinical symptoms, the patients usually die due to respiratory failure caused by chronic bacterial infections [1]. Lungs of patients with CF, due to the presence of viscous mucus already in infancy and early childhood, are colonized by* Staphylococcus aureus* and* Haemophilus influenzae*. These bacteria contribute to the damage of epithelium lining the airways and subsequent colonization with* Pseudomonas aeruginosa* or* Burkholderia cepacia *[2].

Bacterial infections contribute to reducing the airway lumen, the gradual plugging of aging cell, and release of mediators such as anti-inflammatory cytokines, chemokines, proteases, and actin, attracting B and T lymphocytes, especially TH-17 which cause epithelial injury [3].

It is estimated that 54% of the CF population is infected with* P. aeruginosa*, and infection with this bacterium concerns 80% of patients over 25 years old [4].* P. aeruginosa* pigment, pyocyanin, induces rapid and overwhelming apoptosis in neutrophil populations* in vitro*, which is associated with rapid generation of reactive oxygen species (ROS) and the lowering of intracellular cAMP level [5].* S. aureus *infection also contributes to the deterioration of lung function and structure and supports the development of inflammation and oxidative stress (OS), but it is still unclear whether the early infection influences the prognosis of patients [6].

OS is defined as an imbalance between oxidant and antioxidant processes in favor of the former, that leads to excessive levels of ROS and, consequently, damage to biomolecules. Severe OS increases effects of pathological inflammation in patients with CF [7]. OS increases with age of patients, and its harmful effects can be ameliorated by properly mitigated antioxidant supplementation [8]. It has been suggested that antioxidant status of the patients should be monitored closely and antioxidant supplementation should be considered before significant deficiencies develop to ensure optimum antioxidant protection [9, 10].

Increased lipid peroxidation and protein oxidative damage caused by OS had been demonstratedin patients with severe CF [11]. Oxidative modifications of plasma proteinsare reliable and relatively long-lasting markers of OS. Protein carbonyls are mainly generated by the oxidation of several amino acid side chains (e.g., in Lys, Arg, Pro, and Thr) [12]. In addition, carbonyl groups may be introduced into proteins by secondary reaction of the nucleophilic side chains of Cys, His, and Lys residues, with aldehydes (4-hydroxy-2-nonenal, malondialdehyde, or acrolein) produced during lipid peroxidation or with reactive carbonyl derivatives (ketoamines, ketoaldehydes, and deoxyosones) generated as a consequence of the reactions of reducing sugars, or their oxidation products with lysine residues of proteins (glycoxidation reactions), with the eventual formation of the advances glycation end-products (AGEs) [13].

AGEs are a heterogeneous group of compounds with a characteristic fluorescence. Their formation may be accelerated by OS [14]. Advanced oxidation protein products (AOPPs) are formed by oxidants, mainly hypochlorous acid and chloramines, derived from activated neutrophils. Structurally, they are similar to AGEs and have a similar action, inducing pro-inflammatory cytokines and adhesion molecules [15]. Amadori products are stable early glycation products, synthesized by rearranging the initial products of chemical modification of amino groups of proteins by sugars. Amadori modifications are completely structurally distinct compounds from AGEs and require different receptors than those for AGEs. Their formation is also enhanced by OS [13]. Increase in the levels of dityrosine, kynurenine, and* N*′-formylkynurenine and decrease in the level of thiol groups and tryptophan fluorescence are also markers of protein oxidation [16]. Superoxide dismutase (SOD; E.C. 1.15.1.1), catalase (CAT; E.C. 1.11.1.6), and glutathione* S*-transferase (GST; E.C. 2.5.1.18) are endogenous components of the defense system against the harmful effects of free radicals by catalyzing decomposition of superoxide anion (O_2_^•−^), hydrogen peroxide, and inactivate reactive electrophiles, respectively [17]. GST catalyzes conjugation of electrophile xenobiotics and also endogenous compounds, including products of lipid peroxidation, to glutathione, forming less toxic glutathione S-conjugates, actively exported by ABCC transporters [18].

Noninvasive measurement of exhaled nitric oxide (NO) is helpful to assess the inflammation in the airways in different disease entities, including CF [19]. It appears that monitoring inflammation and OS markers in patients with CF may be important for the prevention of disease progression.

This study was aimed at comparing the usefulness of plasma protein oxidation parameters: carbonyl and sulfhydryl group content, AOPP, AGEs, dityrosine,* N*′-formylkynurenine, kynurenine, tryptophan fluorescence, and Amadori products, as well as erythrocyte antioxidant enzyme activities (SOD, CAT, and GST) for assessment of OS in* P. aeruginosa* and* S. aureus* chronically infected pediatric stable CF patients. In addition, concentration of NO was measured in the exhaled air from the lower respiratory tract of the patients.

## 2. Materials and Methods

### 2.1. Ethical Approval

The study was approved by the Regional Medical Council in Rzeszow, Poland. The patients (as well as healthy subjects) and their parents demonstrated their willingness to participate in the study and compliance with its procedures by signing a written informed consent form.

### 2.2. Patients

The inclusion criteria for CF as well as healthy control groups included the age of 9–17 years (pediatric population) and body mass greater than 25 kg. Younger CF patients are usually unable to expectorate sputum derived from secretions in their lower respiratory tract, and therefore oropharyngeal cultures (i.e., upper respiratory tract secretions) are usually performed to detect pathogens. In reality, these cultures detect organisms, including potentially pathogenic ones, present in the throat, not necessarily in the lungs.

Respiratory cultures of 30 patients with CF were analyzed for* P. aeruginosa *and* S. aureus* colonization. Eight patients coinfected with other bacteria were excluded. Finally, 12 pediatric stable CF patients with chronic* P. aeruginosa* (3 males, 9 females, mean age ± SD: 12.8 ± 7.6 years) and 10 patients with chronic* S. aureus* (3 males, 7 females, mean age ± SD: 10.2 ± 3.5 years) lung infection (confirmed by repeated routine microbiological testing) were recruited at the time of a routine clinic appointment for stable CF patients (≥1month from the most recent exacerbation). A control group of 11 healthy subjects (5 males, 6 females, mean age ± SD: 11.3 ± 4.5 years) were recruited from among outpatients of the Provincial Hospital Number 2 in Rzeszow, Poland. Characteristics of the patients are presented in Table 1.

The inclusion criteria for CF stable patients were as follows: chronic pulmonary infection by* P. aeruginosa *or* S. aureus*, defined as the continuous presence of this microorganism in sputum over 1 year prior to the study or at least three* P. aeruginosa*-positive cultures, all separated by more than 1 month during the study period. Antimicrobial drug susceptibility testing was performed by agar dilution according to the methodology of the Clinical and Laboratory Standards Institute [20]. Other inclusion criteria were as follows: forced expiratory volume in the first second (FEV_1_) greater than 35% of predicted, stable pulmonary disease as defined by both clinical impressions and no hospitalizations in the 30 days prior to screening. The respiratory function tests were performed using a standard spirometry device (Lungtest 10000 MES SJ, Cracow, Poland). All children in the control group had normal pulmonary function tests.

Regarding the healthy control group, the inclusion criteria were as follows: no family history of CF or any chronic disease; no history of allergic symptoms; no clinically significant abnormalities as determined by medical history, physical examination, blood chemistry assessments, and hematologic assessments including complete blood count; no use of medication with anti-inflammatory effect (aspirin, other nonsteroidal anti-inflammatory drugs, corticosteroids, and macrolide antibiotics) for 30 days before study; no use of any antioxidants vitamins; and no contraindications to the procedures in this study.

Subjects did not enter the study if they had significant liver disease as defined by clinical findings of portal hypertension or cirrhosis or liver enzymes greater than twice the upper limits of normal values or had participated in another interventional clinical trial within 30 days of screening. Exclusion criteria were also as follows:* Burkholderia cepacia complex* isolated from the respiratory tract at screening or within 2 years of screening; nontuberculous mycobacteria within 2 years of screening or acid-fast bacillus smear positive at screening; use of intravenous antibiotics, quinolones, or other oral antibiotics within 14 days of screening; and use of systemic corticosteroids (≥20 mg of prednisone daily) within 30 days of screening or intake of tobramycin solution for inhalation.

All CF patients had pancreatic insufficiency and were receiving pancreatic enzyme-replacement therapy (Creon 25000, Solvay Pharmaceutical Inc., Marietta, Georgia, USA).CF pediatric patients were also treated with recombinant human DNase I (Pulmozyme, Genentech Inc., San Francisco, California, USA; one 2.5 mg ampoule inhaled once daily using a nebulizer), fat-soluble vitamins in the form of ADEK tablets (Scandipharm, Birmingham, Alabama, USA), supplemental nutrition drinks (Nutrison Protein Plus, Nutricia, Poland), and 3–10% sodium chloride inhalation 3-4 times daily. Participants infected with* P. aeruginosa* who weighed less than 40 kg were instructed to take azithromycin (@visors, Belgium) 1 tablet (250 mg tablets) 3 days a week (Monday, Wednesday, and Friday) and participants who weighed 40 kg or more were instructed to take 2 tablets on the same 3 days per week for 6 months. Administration of this drug was discontinued if a participant had an allergic reaction, a life-threatening adverse event, not including hospitalization for a pulmonary exacerbation, and an adverse event that was considered intolerable by the study team or research participant or if nontuberculous mycobacteria grew from a sputum sample obtained at screening [21]. Maintenance azithromycin therapy in patients with CF leads to macrolide resistance in nearly all* S. aureus* carriers. Pulmonary function improvement after initiation of azithromycin therapy seems to be temporary and appears not to be related to macrolide resistance of* S. aureus* [22].

### 2.3. Biochemical Methods

#### 2.3.1. Chemicals

All basic reagents were from Sigma-Aldrich Company (Poznan, Poland) unless indicated otherwise. All reagents used were of analytical reagent grade.

#### 2.3.2. Blood Sampling

5 mL samples of venous blood from CF stable patients and healthy controls were collected using K_3_-EDTA as the anticoagulant in Sub-Carpathian Center of Pulmonology and Allergology in Rzeszów, Poland. Blood was centrifuged (2000 ×g, 10 min, 4°C) and plasma was aspirated, frozen at −70°C, and used for further assays. The samples were stored for no longer than 2 months and were thawed at room temperature only once at the time of analysis. The suspension of erythrocytes was washed three times with 4 volumes of phosphate buffered saline (PBS; 1 tablet of PBS/100 mL H_2_O) per 1 volume of suspension. The suspension was diluted with PBS at a ratio of 1 : 4 in tubes, frozen at −20°C, and used for further assays.

#### 2.3.3. Clinical Hematological Variables

Clinical hematological variables were estimated in serum samples. 5 mL samples of venous blood from studied CF patients and healthy controls were collected into serum-separating tubes and immediately centrifuged to isolate serum.

Serum triglyceride, glucose, total protein level, C-reactive protein (CRP) (an acute phase protein) concentration, and amylase activity were measured using the dry chemistry immunological method in a VITROS 250 analyzer (Ortho Clinical Diagnostics, Johnson and Johnson, USA). Mean cell volume (MCV), hematocrit (HCT), hemoglobin (Hb), red blood cell (RBC) count, and white blood cell (WBC) count were measured in blood using an ADVIA hematology analyzer (model 2120).

#### 2.3.4. Protein Carbonyl Assay

The content of protein carbonyl was estimated using a modified method of Reznick and Packer [23]. Firstly, to a tube containing 800 *μ*L of 10 mM 2,4-dinitrophenylhydrazine (DNPH) in 2 M HCl, 40 *μ*L of plasma was added. To another tube, containing 800 *μ*L of 2 M HCl, 40 *μ*L of the same plasma was added. The tubes were left at room temperature in the dark for 1 h and were vortexed every 15 min. After incubation, 1 mL of 20% trichloroacetic acid (TCA) was added to each sample; the samples were incubated for 10 min on ice, and the tubes were centrifuged (3000 ×g, 22°C, 3 min). The supernatant fluid was discarded and another wash was performed using 800 *μ*L of 10% TCA solution. The pellets were washed three times with 800 *μ*L of ethanol-ethyl acetate to remove free DNPH and lipid contaminants. The pellets were allowed to dry and then were dissolved in 400 *μ*L of 6 M guanidine hydrochloride solution. The insoluble material was removed by additional centrifugation (3000 ×g 22°C, 3 min). 200 *μ*L-aliquots of the supernatants were applied to a 96-well plate and the absorbance was measured at a wavelength of 370 nm. The results were expressed as nmoles/mg protein, assuming absorption coefficient of 21.0 mM^−1^ cm^−1^ and considering the thickness of the fluid layer in the microplate.

#### 2.3.5. AGE Assay

AGEs were estimated by assessing the formation of glucose-derived fluorescence, termed glycophore, using the spectrofluorimetric method in plasma diluted 1 : 50 with PBS according to Henle et al. [24] and Münch et al. [25] at the excitation and emission wavelengths of 325 and 440 nm, respectively; 150 *μ*L of the diluted plasma was applied to a 96-well plate.

#### 2.3.6. AOPP Assay

Advanced oxidation protein products (AOPPs) were estimated using the method of Witko-Sarsat [15]. 200 *μ*L of plasma diluted 1 : 5 with PBS was applied to the 96-well plate and 20 *μ*L of acetic acid and 10 *μ*L of 1.16 M potassium iodide were added to each well. The absorbance was measured at a wavelength of 340 nm against a blank sample containing 200 *μ*L of PBS, 20 *μ*L of acetic acid, and 10 *μ*L of 1.16 M potassium iodide. Calibration curve was prepared using chloramine-T at concentrations of 0–100 *μ*mol/L by applying 200 *μ*L chloramine-T, 20 *μ*L acetic acid, and 10 *μ*L of 1.16 M potassium iodide to the plate. AOPP concentration was expressed as nmoles of chloramine-T equivalents/mg protein.

#### 2.3.7. Protein Assay

The protein concentration was estimated using the method of Lowry et al. [26] Briefly, 250 *μ*L of the Lowry reagent (formed by mixing 30 mL of 2% Na_2_CO_3_ in 0.1 M NaOH, 0.6 mL of 5% C_4_H_4_O_6_KNa·4H_2_O, and 0.6 mL of 2% CuSO_4_) was applied to a 96-well plate. Then 50 *μ*L of plasma was applied to each well, mixed, and incubated at room temperature for 10 min. Finally, 25 *μ*L of the Folin-Ciocalteu reagent was added, mixed, and incubated at room temperature for 30 min. The absorbance was measured at a wavelength of 750 nm.

#### 2.3.8. Characterization of Amadori Product by the NBT Assay

The content of Amadori product was estimated using the method of Johnson et al. [27], with Nitro Blue Tetrazolium (NBT). 100 *μ*L aliquots of plasma were added to wells of a 96-well plate, followed by 100 *μ*L of the NBT reagent (250 *μ*mol NBT/L of 0.1 M carbonate buffer, pH 10.35) and the plate was incubated at 37°C for 2 h. The absorbance was measured at a wavelength of 525 nm. Amadori products were estimated using an extinction coefficient of 12,640 M^−1^ cm^−1^ for monoformazan [28].

#### 2.3.9. Content of Tryptophan, Dityrosine, Kynurenine, and* N*′-Formylkynurenine

The content of tryptophan, dityrosine, kynurenine, and* N*′-formylkynurenine was estimated on the basis of their characteristic fluorescence at the wavelengths of 295/340 nm 330/415 nm, 365/480 nm, and 325/434 nm, respectively, [29] by measuring the fluorescence of 150 *μ*L of plasma diluted 1 : 50 with PBS in a 96-well plate.

#### 2.3.10. Content of Thiol Groups

The content of thiol groups was estimated by the method of Ellman [30]. In each well of a 96-well plate, 20 *μ*L of plasma and 2 *μ*L of 5,5′-dithiobis-(2-nitrobenzoic acid) (DTNB; 10 mg/mL of 0.1 M phosphate buffer, pH 8.0) were added to 100 *μ*L 0.1 M phosphate buffer, pH 8.0. The samples were incubated for 1 h in the dark at 37°C and the absorbance was measured at 412 nm against the blank. The thiol group content was calculated on the basis of a standard curve using glutathione as a standard.

#### 2.3.11. Catalase Activity

Catalase activity was determined according to Aebi [31]. To 1 mL of 54 mM H_2_O_2_ in 50 mM phosphate buffer, pH 7.0, and 1.95 mL of 50 mM phosphate buffer, pH 7.0, 50 *μ*L of lysate was added to a cuvette and the decrease in hydrogen peroxide concentration was measured spectrophotometrically at 240 nm, at 25°C during 1 min. The kinetic curves were analyzed using the Cary-WinUV-software. One unit of enzyme activity was defined as the activity required to degrade 1 *μ*mol of hydrogen peroxide in 60 s. Enzyme activity was expressed as units per mg of Hb (U/mg Hb).

#### 2.3.12. Glutathione* S*-Transferase Activity

GST activity was determined by the method described by Habig et al. [18]. 250 *μ*L of 0.02 M glutathione in 0.1 M phosphate buffer pH 6.5 was added to 750 *μ*L of 0.1 M phosphate buffer, pH 6.5. 10 *μ*L of 0.1 M 2,4-dinitro-1-chlorobenzene (CDNB) in ethanol and 10 *μ*L of hemolysate had been added to each cuvette. The increase of absorbance was measured at a wavelength of 340 nm for 3 min against a blank. Enzyme activity was expressed as units per gram of Hb (U/g Hb).

#### 2.3.13. Superoxide Dismutase Activity

Superoxide dismutase activity was determined according to the method of S. Marklund and G. Marklund based on the inhibition of pyrogallol autoxidation [32]. Briefly, 500 *μ*L hemolysate, 1000 *μ*L of ethanol, and 600 *μ*L of chloroform were added to a tube containing 3500 *μ*L of cooled distilled water. The samples were mixed after addition of each reagent, then shaken for 1 min, and centrifuged (6000 rpm, 10 min, 4°C). The supernatant was transferred to new tubes. 1760, 1600, 1160, and 160 *μ*L of distilled water and 0, 160, 600, and 1600 *μ*L of supernatant were added, respectively, to cuvettes containing 200 *μ*L of 1 M Tris/HCl—0.005 M EDTA, pH 8.0. Samples were incubated at room temperature for 10 minutes; then, 40 *μ*L of 0.01 M pyrogallol in 0.01 M HCl was added to each cuvette. The increase of absorbance was measured at 420 nm for 5 min against the blank. One unit of superoxide dismutase activity is defined as the amount of enzyme that inhibits the rate of pyrogallol autoxidation by 50%.

#### 2.3.14. Hemoglobin Assay

Hemoglobin (Hb) concentration was estimated on the basis of Drabkin's method [33]. 20 *μ*L of the hemolysate was added to 5000 *μ*L of the Drabkin reagent (0.03% K_3_[Fe(CN)_6_], 0.1% NaHCO_3_, 0.005% KCN), incubated for 15 minutes; then, the absorbance was measured at a wavelength of 540 nm against a blank.

All the spectrophotometric analyses were done using a Varian Cary 50 UV-Vis spectrophotometer (Varian Inc., Cary, NC, USA). Fluorescence was read using an Infinite 200 PRO multimode reader (Tecan Group Ltd., Switzerland).

#### 2.3.15. Measurement of Nitric Oxide

Measurement of nitric oxide in the exhaled bronchial air (eNO) was performed according to American Thoracic Society recommendations [19] using an Hyp'Air Fe_NO_ electrochemical analyzer (MediSoft, Belgium). Patients exhaled through disposable mouthpieces at a constant flow of 50 mL/s for 6 seconds. Measurement of NO was carried out three times for each patient, with subsequent measurements being properly averaged; the results are shown in the ppb (parts per billion) units.

#### 2.3.16. Statistical Analysis

Data are given in the form of arithmetic mean values and standard deviations. Differences between means were analyzed using Kruskal-Wallis test with Tukey's post hoc analysis. The statistical analysis of the data was performed using StatSoft, Inc. (2011), STATISTICA, version 10 (http://www.statsoft.com/).

## 3. Results

Clinical hematological variables were not different between studied groups of CF patients and control subjects (Table 1). The mean values were within the reference range.

Reaction of glucose (and other reducing sugars/aldehydes) with amino groups of proteins leads to formation of products showing specific fluorescence (glycophore). Fluorimetric estimation of the glycophore content of blood serum proteins demonstrated a significant increase in the value of this parameter in* P. aeruginosa* and* S. aureus* infected CF patients versus healthy controls (4.22 ± 0.91 and 4.19 ± 1.04 versus control 3.18 ± 0.53 fluorescence units (FU)/mg protein; *P* < 0.05) (Table 2).

The most commonly used marker of protein oxidation is the formation of carbonyl groups, mainly glutamic semialdehyde (formed from arginine and proline) and aminoadipic semialdehyde (formed by lysine oxidation). Elevated carbonyl group content was noted in* P. aeruginosa* and* S. aureus* infected CF patients (1.9 ± 0.64, 1.87 ± 0.45 versus control 0.94 ± 0.19 nmol/mg protein; *P* < 0.05).

Products of proteins oxidative modifications include dityrosine, kynurenine, and* N*′-formylkynurenine. Glycation of proteins leads to fructosamine-type adducts, which are often assayed as a measure of the degree of glycation. The plasma protein content of kynurenine was significantly increased only in* S. aureus* infected CF patients with respect to control (4.91 ± 1.22 versus 3.89 ± 0.54 FU/mg protein; *P* < 0.05). No differences in the values of other parameters were seen between controls and CF patients with chronic* P. aeruginosa* or* S. aureus* infection.

Oxidative damage to proteins leads, i. a., to destruction of tryptophan residues. There was no significant difference between plasma tryptophan fluorescence in the studied groups of CF patients.

CAT and SOD activities in CF children were at the control level. GST activity was significantly increased in the pediatric CF patients infected with* P. aeruginosa *as well as* S. aureus* versus control subjects (2.51 ± 0.88 and 2.57 ± 0.79 U/g Hb versus 0.77 ± 0.16 U/g Hb; *P* < 0.05).

Noninvasive measurement of NO concentration is helpful to assess the inflammation in the airways of various disease entities, including CF. In the present study, there was no significant difference in the concentration of exhaled NO from lower respiratory tract (eNO) between CF patients infected with* P. aeruginosa* and* S. aureus* in comparison with healthy control.

## 4. Discussion

In CF, chronic infection with bacterial and nonbacterial pathogens combined with profound airway inflammation results in progressive suppurative lung disease, respiratory compromise, and premature death. Paradoxically, these pathogens survive and multiply within the CF lung, despite the presence of an abundant neutrophilic infiltrate, indicating that there is significant impairment of the normal bactericidal mechanisms operating within this environment. The chronic* P. aeruginosa* lung infection is responsible for 90% of the morbidity of patients with CF because of damage of the airways and gradual deterioration of the lung function. The contribution of* S. aureus* to CF lung disease is still less clear.

One hallmark of the CF airway disease is the persistent massive recruitment of polymorphonuclear leucocytes (PMN) [34]. Analysis of bronchoalveolar lavage (BAL) has shown that the number of PMNs recovered from the lungs of patients with CF is 1,000 times higher than that recovered from the lungs of controls. PMNs release leukocyte proteases, myeloperoxidase, and ROS, which are the main mechanisms of lung tissue damage in CF [35]. Patients with CF frequently exhibit increased oxygen free radical generation from activated neutrophils due to chronic lung inflammation [36]. This mechanism is the main contributor to OS in CF. Another factor is an impaired antioxidant status because of malabsorption of dietary antioxidants in the gut leading to lowered intake and absorption of fat-soluble antioxidants (vitamin E, carotenoids, coenzyme Q-10, main polyunsaturated fatty acids, etc.) and oligoelements (such as Se, Cu, and Zn) that are involved in ROS detoxification by means of enzymatic defenses [37]. The next factor is the inability of cells bearing mutant CF transmembrane conductor regulator proteins to efflux glutathione (GSH), the most abundant cellular antioxidant, into the extracellular milieu. Thus, the imbalance between high ROS production and impaired antioxidant systems explains the oxidative stress in CF [34].

Results of this study demonstrate oxidative damage to plasma proteins in CF stable patients chronically infected with* P. aeruginosa* or* S. aureus* in relation to healthy subjects, as evidenced by elevated carbonyl group levels. These findings are in agreement with previous reports. Similar level of carbonylated protein in CF group (1.61 nmol/mg protein) was reported by Brown et al. [38]. Back et al. [9] also observed increased plasma protein carbonyl levels in patients with CF older than 18 years. Winterbourn et al. demonstrated that patients with acute pancreatitis had significantly increased plasma levels of protein carbonyls, which were related to disease severity, thus confirming that this protein modification could be a useful plasma marker of oxidative injury [39].

We noted significantly increased levels of glycophore in plasma proteins in CF patients chronically infected with* P. aeruginosa* or* S. aureus *versus healthy controls. The reasons for this enhanced plasma protein glycoxidation are intriguing. No increased glucose levels were found in the patients; our working hypothesis is that oxidative stress (evidenced by elevated carbonyl group levels) occurring in CF promotes enhanced glycoxidation. Anyhow, these data suggest that the glycophore level may be useful for monitoring OS in CF as a parameter easily assayable in a clinical laboratory.

Reports on enzymatic antioxidant status in CF are inconsistent. Copper-zinc superoxide dismutase (SOD) activity has been reported to be increased, decreased, or unchanged in CF compared with controls [10]. In this study, we found that the activity of SOD in red blood cells was unchanged in both groups of CF patients chronically infected by bacteria. Consistent with previous studies [10], we found that the red blood cell CAT activity was also unchanged. Unchanged activities SOD as well as CAT in this study could be connected with the fact that our CF stable pediatric patients were treated with fat-soluble vitamins in the form of ADEK tablets (Scandipharm, Birmingham, Alabama, USA) and supplemental nutrition drinks (Nutrison Protein Plus, Nutricia, Poland). Our previous study showed that AquADEKs attenuated selected OS markers in pediatric patients with CF [10]. Lezo et al. also reported that CF patients showed elevated OS markers even in stable clinical conditions and with plasma antioxidants within the normal range [8].

The lack of changes in activities of vital erythrocyte antioxidant enzymes is in line with the apparent lack of changes of hematological parameters in the patients. Apparently, the local oxidative stress in the lungs has a limited effect on the blood, confined mainly to plasma proteins which temporarily may be exposed to the oxidative environment of the CF lungs.

In our study, increased GST activity was noted in* P. aeruginosa* and* S. aureus* infected CF. This result is in contrast with that of Feuillet-Fieux et al. [40] who found no differences in the level of GST activity in blood between children with CF and healthy group. GST biosynthesis is upregulated by activation of Nrf2 factor by oxidative/electrophile stress [41]. I. a., pyocyanin, a* P. aeruginosa* pigment, may activate Nrf2 [42].

Our study does not point to significant differences between the* S. aureus* and* P. aeruginosa* infected patients, both in clinical hematologic variables and indices of oxidative protein modifications, with the exception of kynurenine level which was increased only in the patients infected with* S. aureus* suggesting a somewhat higher intensity of oxidative stress. Generally, however, these results suggest that infection with both bacteria triggers oxidative stress resulting in detectable protein modifications.

NO is produced within the respiratory tract and can be detected in exhaled bronchial and nasal air. NO is a highly reactive molecule with important antimicrobial and anti-inflammatory properties. Its concentration varies in specific diseases, being elevated in patients with asthma and bronchiectasis, but decreased in primary ciliary dyskinesia. There are conflicting data on NO levels in CF, which are reported to be unexplained as either decreased or normal [43].

Numerous studies show decreased NO in exhaled air from patients with CF [44]. Thomas et al. [45] also reported that eNO and nNO levels were reduced in patients with CF and there was not any relationship between eNO and patients' genotype. Robroeks and colleagues demonstrated that, in CF, eNO levels were significantly lower compared with controls (3.3 ± 0.3 pg/mL, 2.2 ± 0.2 *μ*M, 10.0 ± 1.2 p.p.b. versus 2.6 ± 0.2 pg/mL, 1.4 ± 0.1 *μ*M, 15.4 ± 1.4 p.p.b. resp.) [46]. Recently, Fila et al. noted lower exhaled breath condensate nitrate concentrations in CF patients than in healthy subjects (5.8 versus 14.3 *μ*M, *P* < 0.001) [44]. de Winter-de Groot et al. reported that low nNO levels are associated with* S aureus* colonization in the oropharynx and lower airways [47]. One potential cause of decreased eNO in CF is upregulation of RhoGTPase, a signaling molecule that reduces nitric oxide synthase (NOS2) expression. Upregulation of RhoGTPase may also contribute to the inflammatory response by increasing IL-8 production. This effect may be also caused by retention of a thick layer of mucus, which restricts the gas diffusion, or reduced activity of NOS2. It is not clear whether the decreased activity of the enzyme is associated with the lack of expression of CFTR gene or is formed secondary to infection and inflammation [47]. Zhou et al. suggested, however, that the correlation between the concentration of eNO and pulmonary function is not sufficient to consider eNO results as a marker of CF [48].

Our results showed that eNO did not differ significantly between CF patients chronically infected with* P. aeruginosa* or* S. aureus* in relation to healthy children. Similarly, Ho et al. and Walker et al. noted that there is no elevated bronchial NO in CF [49, 50].

In summary, our results show no difference in the level of exhaled eNO in CF patients, as compared with healthy controls. Increased levels of glycophore and carbonyl groups in plasma proteins and glutathione* S*-transferase activity in erythrocytes of CF patients with bacterial infections evidence oxidative or electrophile stress in the patients. It is also evident that these parameters are the most sensitive markers of OS in CF patients.

## Figures and Tables

**Table 1:** Characteristics of the population studied.

Parameter	Healthy subjects	*Pseudomonas aeruginosa *	*Staphylococcus aureus *
Demographics			
*n*	11	12	10
Sex (men/women)	5/6	3/9	3/7
Age at enrollment (y)	11.25 ± 4.5	12.78 ± 7.56	10.17 ± 3.54
Height (m)	1.33 ± 0.13	1.4 ± 0.25	1.33 ± 0.22
Weight (kg)	29 ± 7.89	37.44 ± 14.1	34.13 ± 13.63
Body mass index (kg/m^2^)	16.7 ± 1.45	18.71 ± 2.89	18.59 ± 3.75
Genetics			
ΔF508/ΔF508	—	8	1
Unknown	—	0	3
Clinical hematologic variables			
WBC (×10^3^ cells/*μ*L) (reference range, 5.5–15.5)	9.88 ± 1.79	11.63 ± 3.97	9.66 ± 1.88
RBC (×10^6^ cells/*μ*L) (reference range, 3.7–5.3)	4.73 ± 0.33	4.56 ± 0.25	4.67 ± 0.29
Neutrophil ((×10^3^ cells/*μ*L) (reference range, 1.8–8)	4.24 ± 1.09	4.59 ± 2.04	4.33 ± 1.15
Lymphocyte (%) (reference range, 25–50)	45.8 ± 5.66	42.8 ± 9.2	38.52 ± 6.31
Monocyte (%) (reference range, 0–11)	6.2 ± 1.72	6.48 ± 2.08	9.87 ± 4.19
Eosinophil (%) (reference range, 1–5)	2.54 ± 0.83	2.64 ± 1.87	2.8 ± 1.5
Basophil (%) (reference range, 0–1.5)	0.57 ± 0.21	0.7 ± 0.43	0.48 ± 0.33
HCT (%) (reference range, 35–45)	35.8 ± 1.85	38.93 ± 2.2	39.93 ± 3.46
MCV (fL) (reference range, 76–90)	76.96 ± 2.74	83.58 ± 4.59	83.12 ± 6.74
Hb (g/dL) (reference range, 11.5–14.5)	13.1 ± 0.75	13.15 ± 0.45	13.67 ± 1.19
Serum biochemical parameters			
C-reactive protein (mg/L) (reference range, 0–10)	3.77 ± 0.94	4.69 ± 1.72	4.5 ± 2.43
Protein (g/dL) (reference range, 6–8)	6.62 ± 0.35	6.87 ± 0.73	7.19 ± 0.48
Bilirubin (*μ*mol/L) (reference range, 0–17.1)	5.83 ± 0.78	4.38 ± 0.64	6.57 ± 1.53
Glucose (mmol/L)) (reference range, 3.6–6.1)	5.94 ± 0.35	5.08 ± 0.73	5.05 ± 1.24
Aspartate transaminase(AST) (U/L) (reference range, 1–35)	28.37 ± 2.88	27 ± 4.29	32.83 ± 12.12
Alanine aminotransferase(ALAT) (U/L) (reference range, 1–34)	29.63 ± 4.6	26.33 ± 5.57	32.17 ± 15
Gamma-glutamyltransferase (GGTP) (U/L) (reference range, 1–36)	27.4 ± 8.57	23 ± 10.37	14.5 ± 5.96
Alkaline phosphatase(ALP) (U/L) (reference range, 110–369)	258.6 ± 57.2	249.17 ± 61.47	262 ± 98.42
Cholesterol (mg/dL) (reference range, 0–200)	103.2 ± 6.7	133 ± 20.43	114.83 ± 25.01
High-density lipoprotein (mg/dL) (reference range, 0–40)	36.8 ± 2.74	48.5 ± 7.14	41.17 ± 10.96
Low-density lipoprotein (mg/dL) (reference range, 0–180)	70.33 ± 8.4	80 ± 16.79	65.67 ± 9.61
Triglycerides (mg/dL) (reference range, 60–160)	81.9 ± 23.7	95.5 ± 48.79	74.17 ± 46.42
Sodium (mmol/L) (reference range, 135–145)	136.3 ± 1.38	138.5 ± 2.59	138.33 ± 1.97
Potassium (mmol/L, reference range, 3.5–5)	4.01 ± 0.17	4.09 ± 0.2	4.11 ± 0.25
Urea (mmol/L) (reference range, 2.5–6.7)	3.87 ± 0.15	4.28 ± 0.43	3.34 ± 1.02
Creatinine (*μ*mol/L) (reference range, 60–130)	72.7 ± 8.39	36.33 ± 14.61	38 ± 10.89
Pulmonary function			
FEV_1_ (% predicted)	96.5 ± 12.08	71 ± 8.66	94.17 ± 11.39
FEV_1_/FVC (%)	107.5 ± 5.93	79.67 ± 20.01	89.56 ± 17.79

Data are shown as means ± SD. WBC indicates white blood cell; HCT: hematocrit; MCV: mean cell volume.

**Table 2:** Clinical characteristics and oxidative stress markers of the patients studied.

Parameter	Healthy subjects	*Pseudomonas aeruginosa *	*Staphylococcus aureus *
Relative fluorimetric results			
AGE (a.u./mg protein)	3.18 ± 0.53 (2.57–3.81)	4.22 ± 0.91* (3.34–5.89)	4.19 ± 1.04* (3.5–5.28)
Dityrosine (a.u./mg protein)	2.97 ± 0.51 (2.29–3.38)	3.84 ± 1.02 (2.88–5.22)	3.66 ± 1.18 (3.09–5.28)
Formylkynurenine (a.u./mg protein)	3.17 ± 0.58 (2.49–3.83)	4.24 ± 1 (3.21–5.87)	4.17 ± 1.1 (3.50–5.47)
Kynurenine (a.u./mg protein)	3.89 ± 0.54 (3.55–4.51)	4.26 ± 1.1 (3.36–5.8)	4.91 ± 1.22* (3.24–5.91)
Tryptophan (a.u./mg protein)	604.5 ± 59.06 (544.2–658.27)	461.21 ± 132.79 (371.66–497.16)	456.82 ± 142.35 (385.38–516.86)
Modification of proteins			
Carbonyl protein (nmol/mg protein)	0.94 ± 0.19 (0.76–1.15)	1.9 ± 0.65* (0.96–2.93)	1.87 ± 0.46* (1.28–2.33)
Thiol groups (nmol/mg protein)	19.67 ± 0.92 (18.6–20.49)	17.46 ± 3.87 (12.6–19.05)	18.35 ± 3.83 (11.19–19.83)
AOPP (nmol/mg protein)	160.03 ± 17.5 (141.09–183.32)	214.85 ± 81.69 (137.18–318.19)	186.97 ± 77.6 (139.71–293.77)
Amadori products (nmol/mg protein)	1577.45 ± 115.89 (1416.4–1675.38)	1774.22 ± 692.99 (1234.74–2878.33)	1608.45 ± 166.68 (1351.38–1762.19)
Activity of erythrocyte enzymes			
Glutathione S-transferase (U/g Hb)	0.77 ± 0.17 (0.55–0.94)	2.51 ± 0.88* (1.43–3.99)	2.52 ± 0.79* (1.49–3.91)
Catalase (U/mg Hb)	0.37 ± 0.06 (0.29–0.39)	0.29 ± 0.07 (0.19–0.37)	0.3 ± 0.04 (0.22–0.33)
Superoxide dismutase (U/g Hb)	1150.78 ± 110.85 (1056.4–1296.93)	1072.09 ± 325.6 (690.8–1671.34)	1165.71 ± 195.2 (859.38–1387.81)
Total antioxidant capacity (nmol/mg protein)	17.84 ± 1.83 (14.22–18.44)	24.49 ± 5.36 (18–30.67)	19.45 ± 2.06 (17.84–21.77)
Level of nitric oxide eNO (ppb)	9.68 ± 4.7 (5.3–22.4)	11.19 ± 5.21 (6.4–20.6)	9.8 ± 3.97 (5.6–16.8)

Data are shown as means ± SD (range).

*P < 0.05.
